# Leucine-Rich α2-Glycoprotein Is a Novel Biomarker of Neurodegenerative Disease in Human Cerebrospinal Fluid and Causes Neurodegeneration in Mouse Cerebral Cortex

**DOI:** 10.1371/journal.pone.0074453

**Published:** 2013-09-18

**Authors:** Masakazu Miyajima, Madoka Nakajima, Yumiko Motoi, Masao Moriya, Hidenori Sugano, Ikuko Ogino, Eri Nakamura, Norihiro Tada, Miyuki Kunichika, Hajime Arai

**Affiliations:** 1 Department of Neurosurgery, Juntendo University Graduate School of Medicine, Tokyo, Japan; 2 Department of Neurology, Juntendo University Graduate School of Medicine, Tokyo, Japan; 3 Laboratory of Genome Research, Research Institute for Diseases of Old Age, Juntendo University Graduate School of Medicine, Tokyo, Japan; 4 Division of Biomedical Imaging Research, Juntendo University Graduate School of Medicine, Tokyo, Japan; Cleveland Clnic Foundation, United States of America

## Abstract

Leucine-rich α2-glycoprotein (LRG) is a protein induced by inflammation. It contains a leucine-rich repeat (LRR) structure and easily binds with other molecules. However, the function of LRG in the brain during aging and neurodegenerative diseases has not been investigated. Here, we measured human LRG (hLRG) concentration in the cerebrospinal fluid (CSF) and observed hLRG expression in post-mortem human cerebral cortex. We then generated transgenic (Tg) mice that over-expressed mouse LRG (mLRG) in the brain to examine the effects of mLRG accumulation. Finally, we examined protein-protein interactions using a protein microarray method to screen proteins with a high affinity for hLRG. The CSF concentration of hLRG increases with age and is significantly higher in patients with Parkinson’s disease with dementia (PDD) and progressive supranuclear palsy (PSP) than in healthy elderly people, idiopathic normal pressure hydrocephalus (iNPH) patients, and individuals with Alzheimer’s disease (AD). Tg mice exhibited neuronal degeneration and neuronal decline. Accumulation of LRG in the brains of PDD and PSP patients is not a primary etiological factor, but it is thought to be one of the causes of neurodegeneration. It is anticipated that hLRG CSF levels will be a useful biomarker for the early diagnosis of PDD and PSP.

## Introduction

The activation of inflammatory pathways in the brain is increasingly emphasized as a major risk factor for the development and progression of neurodegenerative disease [1-3]. Long-term use of anti-inflammatory drugs reduces the risk of Alzheimer’s disease (AD) and Parkinson’s disease (PD) [4-6], and animal studies have provided additional support for this hypothesis [7-9]. Furthermore, Berchtold et al. [10] reported that neuroinflammation is present in the brain long before cognitive decline, and immune activation is a highly prominent feature of normal brain aging.

Leucine-rich α2-glycoprotein (LRG) was first identified as a trace protein in human serum in 1977 [11]. This 24-amino acid consensus sequence, termed the leucine-rich repeat (LRR), has since been identified in a large family of proteins [12]. The LRR family is diverse, and its members have been shown to be involved in protein-protein interaction, signal transduction, and cell adhesion and development [13]. Shirai et al. [14] reported that LRG expression was enhanced by acute inflammation induction in mice. Therefore, like C-reactive protein (CRP), LRG might serve as a diagnostic biomarker in some inflammatory conditions.

Gait disturbance and dementia are present in numerous neurodegenerative disorders, including idiopathic normal pressure hydrocephalus (iNPH), PD with dementia (PDD), dementia with Lewy bodies (DLB), progressive nuclear palsy (PSP), and AD. Sometimes it is difficult to correctly diagnose these diseases, but cerebrospinal fluid (CSF)-based diagnosis of AD using the biomarkers amyloid β42 (Aβ42), total tau protein (t-tau), and hyperphosphorylated tau (p-tau) has been reported as useful [15]. However, the other neurodegenerative diseases described above lack disease-specific CSF biomarkers. Of these, iNPH is the only treatable dementia; symptoms can remit following a shunt procedure. iNPH is often comorbid with PD, PSP, or AD, but early diagnosis is difficult based on neurological symptoms and neuroimaging. Furthermore, even if neurological symptoms show improvement following a shunt procedure, a gradual decline in neurological symptoms has been observed with clear onset of co-morbid neurodegenerative diseases after several years [16]. As such, the development of CSF biomarkers to identify iNPH and other neurodegenerative diseases is an extremely important issue.

To examine the function of LRG in the brain in the context of aging and neurodegenerative diseases, we first measured human hLRG concentration in the CSF and observed hLRG expression in the cerebral cortex of autopsied brains. We then generated conditional Tg mice that overexpressed mouse mLRG in the brain to examine the effects of mLRG accumulation in the cerebral cortex. Finally, to examine protein-protein interactions, we used a protein microarray method to screen proteins with a high affinity for hLRG.

## Materials and Methods

### Subjects and study design

This study was conducted over a period of 6 years between January 2006 and September 2012 on consecutive, unselected admissions to our program for 100 patients with clinically suspected iNPH (63 men, 37 women; mean age 75.8 ± 6.7 years) [17,18]. In addition, 26 elderly individuals (13 men, 13 women; mean age, 68.3 ± 9.8 years) without subjective cognitive impairment or known brain disease, with MMSE scores >25 and who consented to lumbar puncture at the Juntendo University Hospital were included as non-demented NCs. We also included 43 patients (24 men, 19 women; mean age, 73.9 ± 9.7 years) with probable AD in this study. The diagnostic criteria of the National Institute of Neurological Disease and Communicative Disorders and the Stroke/Alzheimer’s Disease and Related Disorders Association (NINCDS/ADRDA) were used to identify AD [19]. For PSP (possible PSP, n = 18), diagnoses were made according to the NINDS-SPSP criteria [20] for PSP. Among the iNPH, AD, and PSP patients, dementia severity was evaluated with the MMSE.

### Ethics statement

All the patients (or their next of kin) provided written informed consent to participate in the study, including CSF protein measurements. Approval for this research was granted by the ethics committee of Juntendo University, Japan. All experiments were carried out with the approval of the institutional animal care committee of Juntendo University.

### CSF samples and biomarker assay

Lumbar puncture was performed in the L3-L4 or L4-L5 interspace. A 10–30 ml CSF sample was collected and gently mixed to avoid gradient effects. CSF samples with >500 erythrocytes/μl were excluded. All CSF samples were centrifuged to remove cells and debris, aliquoted, and stored in polypropylene tubes at -80°C until biochemical analysis. The CSF biomarkers total tau (Tau) and tau phosphorylated at threonine 181 (p-tau) were determined with commercially available ELISAs (Innotest hTau-Ag and Innotest Phosphotau [181P], Innogenetics, Ghent, Belgium). hLRG and Abeta42 levels were also measured using commercially available ELISAs (Immuno Biological Laboratories [IBL], Gunma, Japan). All assays were performed according to the manufacturers’ protocols.

### Human LRG antibody for immunostaining

We produced a custom anti-human hLRG (329) rabbit immunoglobulin (Ig) G Affinity Purify (IBL) custom antibody for immunostaining of the brain.

hLRG-cDNA was transfected into simian CV-1 cells carrying SV40 genetic material (COS, CHO-K1, HEK293) that were harvested by scraping with TNE buffer (10 mM Tris [pH 8.0], 1% NP-40, 150 mM NaCl, 1 mM EDTA) according to the instruction manual for Lipofectamine 2000 (Invitrogen, Carlsbad, CA, USA). Briefly, cells were cultured in Dulbecco’s modified Eagle’s medium (DMEM; Sigma-Aldrich, St. Louis, MO, USA), and then seeded with 10% fetal calf serum in 24-well plates. Then, the media was removed, and plasmid DNA-Lipofectamine 2000 Complex (in Opti-MEM) was prepared and added to wells containing serum-free cells. After 4 h, the medium was changed to DMEM/10% fetal calf serum.

At 48 h after transfection, lysates from hLRG-transfected COS cells were suspended in 50 µl 2× sample buffer (2% SDS, 10% glycerol, 50 mM Tris-HCl [pH 6.8], 100 mM dithiothreitol), and 5-µl aliquots were applied to 12% acrylamide gels for electrophoresis. Next, proteins were transferred to polyvinylidene fluoride membranes (Millipore, Billerica, MA, USA), and nonspecific bands were blocked with 3% milk containing 1% bovine serum albumin (BSA) and 0.05% NaN_3_ in phosphate-buffered saline (PBS) for 2 h at 37°C. Using an electrochemiluminescence blot kit (RPN2106; GE Healthcare, Piscataway, NJ, USA), bands were immunoreacted with the primary antibody anti-hLRG(329) (2 µg/ml) overnight at 4°C followed by horseradish peroxidase-conjugated anti-rabbit IgG (17502, IBL) as the secondary antibody for 1 h at 37°C.

Transfected COS cells, CHO-K1 cells, and HEK293 cells were fixed in 10% formalin/PBS for 10 min at 4°C, followed by acetone/methanol (1:1, v/v) for 10 min at 4°C. For blocking, cells were incubated with 5% normal goat serum/BSA/PBS for 20 min at 4°C. Anti-hLRG (329) rabbit IgG (5 µg/ml) was applied for 120 min at 4°C for the primary antibody reaction, and Alexa Fluor 488 goat anti-rabbit IgG (H+L) was used for the secondary antibody reaction.

### Immunohistochemistry

Paraffin sections from each area of the autopsied 19 human and 31 mouse brains were immunostained with anti-hLRG(329) rabbit IgG (1:20, IBL) or anti-mLRG(138) rabbit IgG (1:20, IBL) and goat IgG (1:100, sc-13914; Santa Cruz Biotechnology, Santa Cruz, CA, USA), anti-tau (phosphor S396) rabbit IgG [E178] (1:500, ab32057; Abcam, Cambridge, UK), anti-NeuN mouse IgG [A60] (1:100, MAB377; Millipore) and anti-neurofilament-L (NF-L) goat IgG [C-15] (1:200, sc-12980; Santa Cruz Biotechnology).

Staining was performed with diaminobenzidine (DAB), followed by treatment with Dako Envision System-Labeled Polymer, HRP (K1491; Dako, Glostrup, Denmark) and Histofine Simple Stain MAX PO (G) (414161; Nichirei Biosciences, Tokyo, Japan) as secondary antibodies.

Sections were viewed under a microscope (E800; Nikon, Tokyo, Japan), and images were captured with a AxioCam HRc CCD camera (Carl Zeiss, Oberkochen, Germany) using AxioVison Rel 4.7 imaging-processing software (Carl Zeiss).

### Generation of CAG-EGFP/mLRG^LoxP^ Tg mice

To examine whether LRG overexpression would induce neurodegeneration, B6D2F1 mice (C57BL/6N×DBA/2N; Charles River Laboratories Japan, Kanagawa, Japan) were used as oocyte and sperm donors for intracytoplasmic sperm injection-mediated transgenesis. Mice were fed ad libitum with a standard diet and maintained in an air-conditioned and light-controlled animal room (23 ± 1°C, 55 ± 5%, 12-h light/12-h dark). All animal experiments were performed in accordance with the guidelines of the Laboratory Animal Experimentation at Juntendo University School of Medicine.

Mouse LRG (mLRG) cDNA (BC030733, Invitrogen) was subcloned into a LoxP-GFP-pA-LoxP, which was excised with *Kpn*I and *Not*I. This *Kpn*I/*Not*I restriction fragment was inserted into the *Eco*RI site of the third exon of the rabbit-globin gene in the expression plasmid pCAGGS, which contains cytomegalovirus enhancer, chicken-actin promoter, β-actin intron, and rabbit -globin poly-A signal [21]. The resulting construct (designated CAG-LoxP-GFP-pA-LoxP-mLRG, 4.72 kbp), isolated from the plasmid backbone by digestion with *Sca*I/*Bam*HI, was dissolved in 10 mM Tris-HCl (pH 7.6)/0.1 mM EDTA and then stored at 4°C until use.

Generated CAG-EGFP/mLRG^LoxP^ transgenic mice were crossed with hGFAP-Cre transgenic mice (B6.FVB-Tg(EIIa-cre) C5379Lmgd/J; Jackson Laboratory, Bar Harbor, ME, USA) [22] for conditional Cre-mediated transgene expression. When crossed with CAG-EGFP/mLRG^Loxp^ transgenic mice, Cre-mediated recombination and specific expression of mLRG occurs in astrocytes, oligodendroglia, ependymal cells, and some neurons in the hybrid mice (CAG-EGFP/mLRG^LoxP^ x hGFAP-Cre).

Genomic DNA from CAG-EGFP/mLRG^LoxP^ transgenic mice was prepared from tail biopsies following standard procedures and used for polymerase chain reaction (PCR) analysis. Oligonucleotides used for detecting the specific 215-base pair PCR product of cytomegalovirus (CMV) were CMV-1F (sense primer) 5′-gggtcattagttcatagcc-3′ and CMV-2R (antisense primer) 5′-ggcatatgatacacttgat-3′. Genotyping of GFAP-Cre transgenic mice was performed by Taq polymerase PCR for the Cre primer set (Cre-S, sense primer 5′-tttgcctgcattaccggtcgatgcaac-3′; Cre-AS, antisense primer 5′-tgcccctgtttcactatccaggttacgga-3′).

### In situ hybridization

Mouse LRG probes were designed and applied according to the QuantiGene ViewRNA protocol by using branched DNA technology (Panomics, Fremont, CA, USA). Five-micrometer-thick paraffin-embedded tissue sections were fixed, permeabilized with protease, and hybridized with oligonucleotides conjugated to alkaline phosphatase. After incubation with FastRed substrate, slides were counterstained with hematoxylin and mounted with coverslips using Permount (Fisher Scientific, Pittsburgh, PA, USA). Images were acquired using a Zeiss AX10 Scope AI, ProgRes digital microscope camera and Mac Capture Pro 2.6.0 software (Jenopik, Jena, Germany).

### Western blot analysis of mLRG expression in mouse brain

Levels of mLRG in the cerebral cortex of Tg and WT mice were determined with immunoblot analyses. The cerebral cortex was dissected from mice after they were lethally anesthetized in accordance with protocols approved by Laboratory Animal Research of Juntendo University. Cerebral tissues were sonicated in lysis buffer (150 mM NaCl, 1mM EDTA, 0.5% Triton X-100, 1% SDS, 10 mM Tris [pH 7.4]) containing 1 mM PMSF, 10 mM NaF, and 1% (v/v) protease and phosphatase inhibitor cocktails (Sigma-Aldrich). The samples were clarified through centrifugation at 20,000× *g*, and protein concentrations were determined using a BCA kit (Pierce/Thermo Scientific, Waltham, MA, USA). Equal amounts of protein were run on 10% Criterion-XT gels (Bio-Rad Laboratories, Hercules, CA, USA) and transferred to nitrocellulose membranes (GE Healthcare). Membranes were probed with mouse anti-LRG antibody followed by fluorophore-conjugated (anti-mouse-Cy3 and anti-rabbit-Cy5) goat secondary antibodies (GE Healthcare). The signals were acquired with a Typhoon fluorescent scanner (GE Healthcare), and the band intensities were quantified using ImageQuant software (GE Healthcare).

### Production of DYKDDDDK-human LRG

The full-length cDNA of the *human LRG* coding region was inserted into the pcDNA3.1/Hygro (-) vector (Invitrogen), adding a DYKDDDDK-tag to its C-terminus and transfected into CHO-K1 cells.

The culture supernatant of stable transfectant was collected and assessed with affinity column chromatography using ANTI-DYKDDDDK M2 Affinity Gel (Sigma-Aldrich). The concentration of the purified protein was measured by Human LRG Assay Kit-IBL (IBL).

### Protein-protein interactions using protein arrays

DYKDDDDK-human LRG was assayed at concentrations of 5 and 50 ng/µl on ProtoArray Human Protein Microarrays v5.0 using the manufacturer’s protocol to identify interactions with human proteins present on the ProtoArray, which contains over 9,000 different human proteins. Interacting proteins were identified using mouse anti-DYKDDDDK antibody followed by Alexa Fluor 647-anti-mouse IgG. Arrays were then washed and scanned with a fluorescence microarray scanner.

### Identification of protein interactors for DYKDDDDK-human LRG

All possible protein interactors were evaluated by their signal characteristics within the array and compared to the negative control assay. A protein was defined as exhibiting a significant interaction if it met the following conditions: the Z-Factor, or signal-to-noise ratio, was >0.5, indicating a signal greater than 2-fold above the noise. The Z-Score, or normalized fluorescent signal, was >2.0 standard deviations above the mean human protein signal on the corresponding array. The Signal Used value was at least 3-fold higher than the corresponding Signal Used value in the negative control assay. The protein had a replicate spot coefficient value <50% on the corresponding array.

### Statistical analysis

All analyses were performed with PASW Statistics software, version 18.0 (SPSS, Inc., Chicago, IL). To assess differences between two groups, Mann-Whitney *U* tests were used, and one-way analysis of variance (ANOVA) followed by Dunnett T3 post hoc tests were used for multiple comparisons. Correlations were evaluated using linear regression analysis (Pearson’s correlation), as well as Spearman’s nonparametric correlation to control for potential contributions secondary to outliers.

A receiver operating characteristic (ROC) curve was used to calculate the relationship between sensitivity and specificity for the disease group vs. healthy or disease controls, and to evaluate the diagnostic performance of the analytes. The optimum cutoff value from the ROC curve was determined by anchoring the sensitivity to 90% to 95%, except in a few cases where sensitivity was below 90% but the sum of sensitivity and specificity was maximal. Values of p < 0.05 were regarded as significant, and values of p < 0.001 were used for all our major findings to minimize errors related to multiple comparisons.

## Results

### Human CSF analysis

#### Effect of age

**Figure 1:**
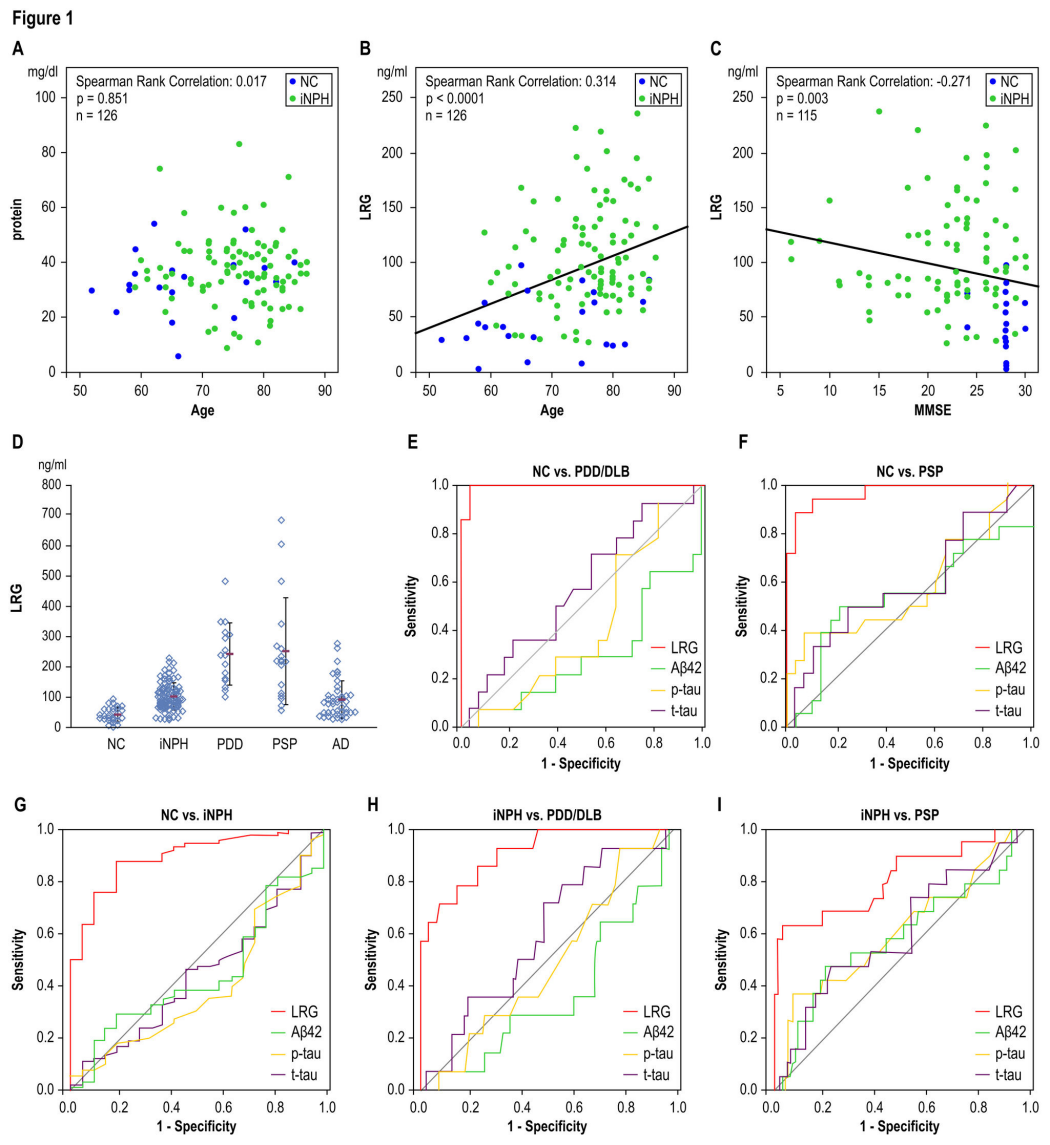
Human CSF analysis. Relationships between age and CSF total protein and LRG levels. (A) CSF total protein level did not correlate with age (r = 0.031, p = 0.731) (B) CSF LRG level significantly correlated with age (r = 0.314, p < 0.0001) using Spearman rank correlation. (C) CSF LRG level reverse correlated with the MMSE score (r = -0.271, p = 0.003) using Spearman rank correlation. LRG levels were significantly higher in the MMSE ≤ 23 group (113.6 ± 70.0 ng/ml) than in the MMSE > 23 group (92.0 ± 58.3 ng/ml), Mann-Whitney U test, p < 0.05). (D) CSF LRG levels tended to be higher in the iNPH groups (106.0 ± 46.7 ng/ml) vs. NC groups (44.0 ± 25.2 ng/ml), Mann-Whitney U test, p < 0.001. Concentrations of LRG in the PDD/DLB (251.5 ± 106.5 ng/ml) and PSP (261.1 ± 182.9 ng/ml) groups were significantly higher than those in the NC, iNPH, and AD groups (95.1 ± 64.4 ng/ml), Mann-Whitney U test, p < 0.001; PD/DLB vs. NC, p < 0.001; PSP vs. NC, p = 0.001; PDD/DLB vs. iNPH, p = 0.021, PSP vs. iNPH; p < 0.001, PDD/DLB vs. AD; p = 0.013, PSP vs. AD). ROC analysis of CSF biomarkers. LRG was the best discriminating biomarker for (E) PDD/DLB patients vs. NC, (F) PSP patients vs. NC, (G) iNPH patients vs. NC, (H) iNPH patients vs. PDD/DLB, and (I) iNPH patients vs. PSP, LRG was the most discriminating biomarker. CSF = cerebrospinal fluid, LRG = leucine-rich α2-glycoprotein. MMSE = Mini-Mental State Examination, iNPH = idiopathic normal pressure hydrocephalus, NC = normal control, PDD = Parkinson disease with dementia, DLB = dementia with Lewy bodies, PSP = Progressive Supranuclear Palsy, AD = Alzheimer disease, Amyloid(1-42) = amyloid beta peptide 1-42, p-TAU = phosphorylated tau, TAU = total tau.

CSF obtained from 26NC and 100iNPH patients was tested for total protein and hLRG levels using a bicinchoninic acid assay (BCA) method and enzyme-linked immnunosorbent assay (ELISA). Notably, hLRG CSF levels significantly increased with age (Spearman rank correlation, r = 0.314, p < 0.0001, n = 126), while concentrations of total protein were stable (r = 0.017, p = 0.851, n = 126) (Figure 1A, B).

#### CSF hLRG level and MMSE score

CSF hLRG concentrations were negatively correlated with the MMSE score (r = -0.271, p = 0.003, n = 115) using Spearman rank correlation (Figure 1C).

#### Group differences of CSF LRG levels

CSF LRG levels tended to be higher in iNPH groups vs. normal control (NC) groups (Table 1, p < 0.001). Concentrations of hLRG in the PDD/DLB and PSP groups were significantly higher than those in the NC, iNPH, and AD groups (p < 0.001, PDD/DLB vs. NC; p < 0.001, PSP vs. NC; p = 0.001, PDD/DLB vs. NPH; p = 0.021, PSP vs. iNPH; p < 0.001, PDD/DLB vs. AD; p = 0.013, PSP vs. AD) (Figure 1D).

**Table 1:** Study Participants Demographics and CSF Marker Levels in Diagnostic Groups.

	NC	iNPH	PDD/DLB	PSP	AD	*p*						
No. of cases	26	100	16	18	43	iNPH/NC	PDD/NC	PSP/NC	iNPH/PD	iNPH/PSP	PD/AD	PSP/AD
Age	68.3±9.8	75.8±6.7	71.2±11.6	74.9±6.6	73.9±9.7							
Gender (F/M)	13/12	37/63	8/8	3/15	19/24							
LRG (ng/ml)	44± 25.2	106±46.7	251.5±106.5	261.1±182.9	95.2±64.4	<0.001	<0.001	<0.001	0.001	0.021	<0.001	0.013
t-tau (pg/ml)	155.9±92.5	149.7±105	163.0±90.2	187.1±108.4	416.7±302	1.000	1.000	0.974	1.000	0.843	<0.001	0.001
p-tau (pg/ml)	29.2±9.1	26.8±13.4	22.1±6.2	34.0±15.5	63.7± 39.3	0.976	0.988	0.931	1.000	0.514	0.001	0.001
Aβ42 (pg/ml)	324±223	317.9±232.7	247.3±221.1	408.8± 250.4	292±244.3	1.000	0.961	0.937	0.933	0.797	0.999	0.641
p-tau/Aβ42	0.10±0.06	0.14± 0.17	0.25± 0.32	0.13±0.12	0.75±1.7	0.590	0.623	0.989	0.885	1.000	0.569	0.232

Data shown are mean + SD. Comparisons were made using one-way ANOVA followed by Dunnett T3 post hoc tests. Conservatively, values with p < 0.001 were regarded as significant to minimize errors related to multiple comparisons. Only the ratios discussed in the text are listed here. NC = non-demented controls, iNPH = idiopathic normal pressure hydrocephalus, PDD/DLB = Parkinson disease with dementia/dementia with Lewy bodies, PSP = progressive supranuclear palsy, AD = Alzheimer’s disease, ANOVA = analysis of variance, CSF = cerebrospinal fluid, LRG = leucine-rich α2-glycoprotein, Aβ42 = amyloid beta peptide 1-42, p-tau = phosphorylated tau, SD = standard deviation, t-tau = total tau.

#### Diagnostic utility of individual biomarkers

The results are shown in Table 2.

**Table 2:** AUC values, sensitivities, specificities, and cutoff points of the Best discriminating parameters for each differential diagnostic test.

Differential Diagnosis	Parameter	AUC	Cutoff	Sensitivity	Specificity
NC vs. PD/DLB	LRG	0.995	115	0.929 (1.00 - 0.857)	0.964 (1.000 - 0.929)
NC vs. PSP	LRG	0.970	79	0.889 (0.944 - 0.833)	0.929 (0.970 - 0.893)
NC vs. iNPH	LRG	0.894	74	0.761 (0.795 - 0.750)	0.909 (0.955 - 0.864)
iNPH vs. PD/DLB	LRG	0.913	134	0.857 (0.929 - 0.786)	0.798 (0.808 - 0.778)
iNPH vs. PSP	LRG	0.808	140	0.684 (0.737 - 0.632)	0.798 (0.808 - 0.788)

Cutoff refers to the selected value of the individual biomarker where the two groups can be separated at the indicated sensitivity and specificity. Values were considered significant at p < 0.001 for all tests. Confidence intervals (95%) are shown for sensitivity and specificity.

For NC vs. PDD/DLB, the pattern of analytes was significantly different between NC and PDD/DLB. Receiver-operating characteristic (ROC) statistics showed that hLRG was superior to the other biomarkers in terms of area under the curve (AUC) (Figure 1E). hLRG concentration was higher on average in the PDD/DLB groups than in the NC group with an excellent diagnostic sensitivity (92.9%) and specificity (96.4%).

For NC vs. PSP, the analyte pattern also differed between NC and PSP; ROC statistics demonstrated that hLRG was superior to any of these biomarkers alone in terms of AUC (Figure 1F). hLRG concentration was higher on average in the PSP groups than in the NC group with an excellent diagnostic sensitivity (88.9%) and specificity (92.9%). For NC vs. iNPH, high sensitivity and specificity were achieved with hLRG (sensitivity, 76.1%; specificity, 90.9%) (Figure 1G).

For iNPH vs. PDD/DLB, when comparing NC and PDD/DLB, high sensitivity and specificity could be achieved with hLRG (sensitivity, 85.7%; specificity, 79.8%) (Figure 1H). For iNPH vs. PSP, as mentioned above, it is often difficult to clinically differentiate between iNPH and Parkinson’s disorders, particularly at the early disease stages, and there is no established laboratory test or biomarker that can assist in differential diagnosis in a regular hospital setting. With CSF hLRG, iNPH patients were differentiated with a sensitivity of 68.4%, and specificity of 79.8% (Figure 1I).

The analyte patterns differed among NC, iNPH, PDD/DLB, and PSP; the ROC statistics showed that LRG was superior to any of the assessed biomarkers alone in terms of AUC.

### Immunostaining of autopsied brains

**Figure 2:**
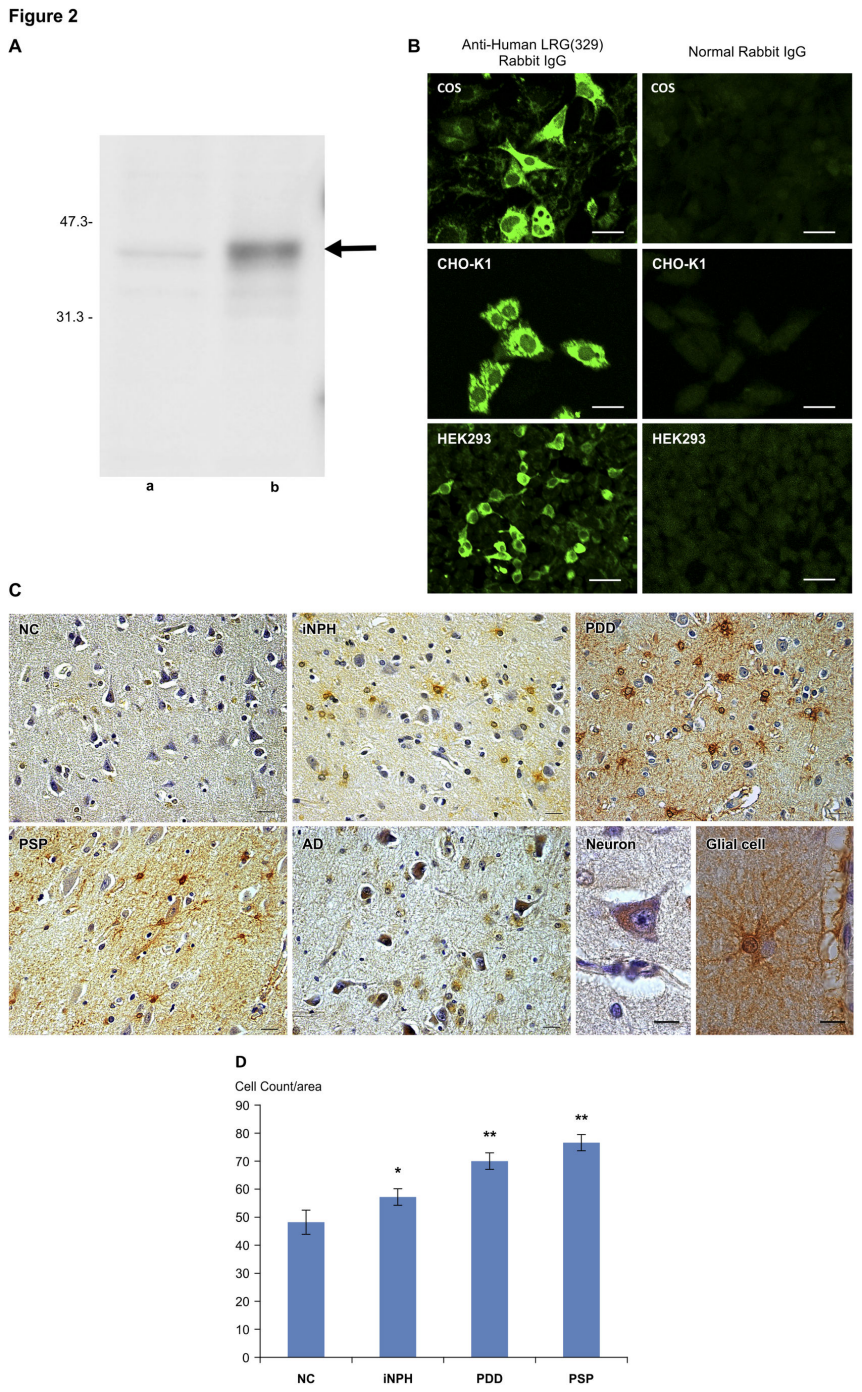
LRG immunostaining of autopsied brains. (A) Western blotting of hLRG-transfected COS cell lysates showed strong expression of a 38-kDa band corresponding to LRG (arrow). **a**: COS cell lysate, **b**: hLRG-transfected COS cell lysates. (B) Immunoreactivity in hLRG-transfected cells was confirmed by immunostaining with anti-hLRG(329) rabbit IgG. COS cells, CHO-K1 cells, HEK293 cells. Scale bar = 20µm. (C) hLRG immunoreactivity in some glial cells and neurons in the internal pyramidal layer and multiform layer of the frontal cortex. Compared to the NC, LRG-immunoreactive cells were increased in iNPH. There was a significant increase in LRG-positive cells in PDD and PSP. With AD, we confirmed strong LRG immunoreactivity in neurons rather than in glial cells. Scale bar = 20µm (NC, iNPH, PDD, PSP and AD) and 10µm (Neuron and Glial cell). NC: 74-year-old male, frontal cortex iNPH: 76-year-old, male, frontal cortex. PDD: 69-year-old, male, frontal cortex. PSP: 65-year-old, male, temporal cortex, glial cell. AD: 82-year-old, male, frontal cortex, neuron. (D) Quantitation of hLRG immunoreactive cells in the cerebral cortex. Stained cells in a 0.05-mm^2^ area of each cortical region were counted by independently by two investigators. The values on the y-axis represent the number of immunoreactive cells and are expressed as the means ± standard deviations for five areas in each case, and the results were analyzed using a one-way ANOVA followed by Dunnett’s test, *p < 0.05, **p < 0.001. Bars, + SD.

Western blotting of hLRG-transfected COS cell lysates showed strong expression of a 38-kDa band corresponding to hLRG (Figure 2A). Immunoreactivity in hLRG-transfected cells was confirmed with an anti-hLRG(329) rabbit IgG (Figure 2B), and this antibody was used to immunostain cerebral cortex sections of autopsied brains.

With NCs (n = 11), we confirmed hLRG immunoreactivity in some glial cells and neurons in the deep layer of the cerebral cortex. Compared to the NC, hLRG-positive glial cells increased with iNPH (n = 2). Furthermore, with PDD (n = 2) and PSP (n = 2), there was a significant increase in the number of glial cells positive for hLRG, and we confirmed immunoreactivity in the protruding glial cell foot processes. We also confirmed hLRG immunoreactivity in neuronal cytoplasm. With AD (n = 2), we confirmed strong hLRG immunoreactivity in neurons rather than in glial cells (Figure 2C). We quantified the number of cells demonstrating a positive immunoreactivity and observed a difference between NC and iNPH with regard to the number of LRG-positive cells (p < 0.05). We also noted a significant increase in the number of LRG-positive cells in PDD and PSP as compared to that in NC (p < 0.001) and iNPH (p < 0.001) (Figure 2D). Importantly, hLRG immunoreactivity in post-mortem cerebral cortex corresponded to the hLRG concentration measured in the CSF.

### Age-related changes in mouse cerebral cortex

**Figure 3:**
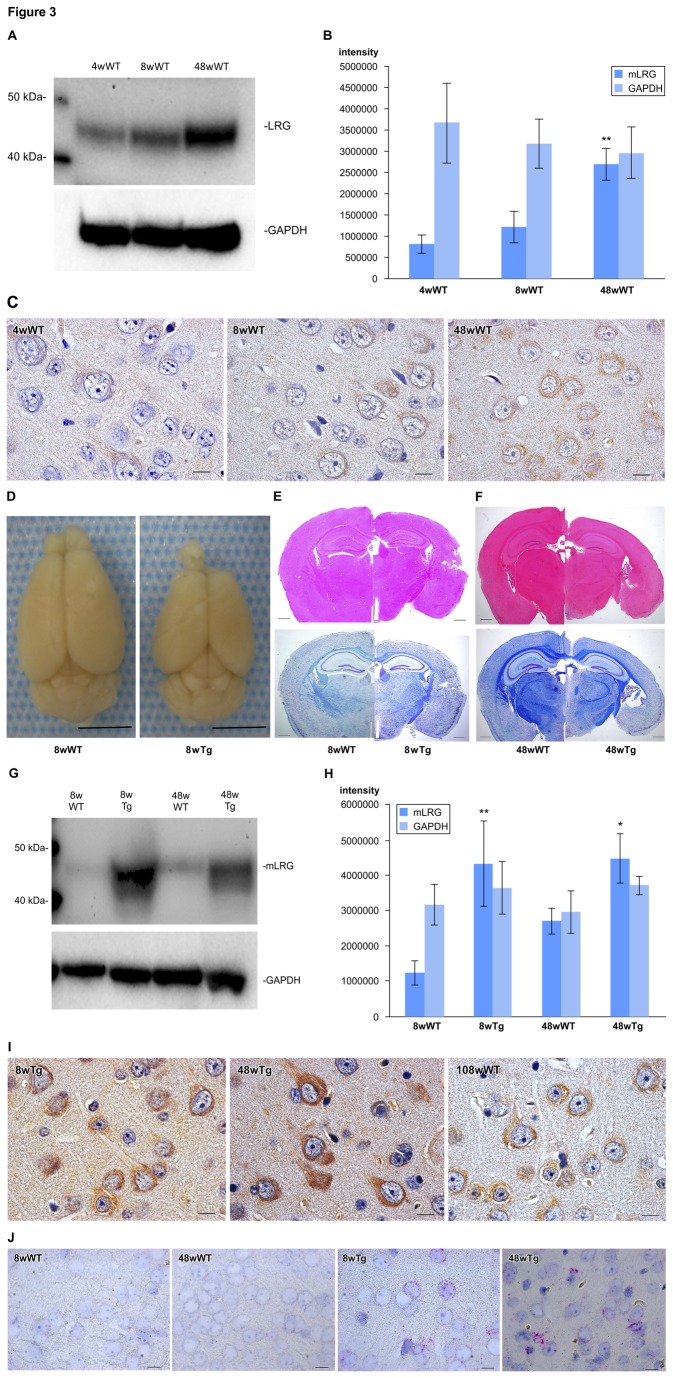
Age-related changes in mouse cerebral cortex. (A) Western blot analysis. Compared to the 4- and 8-week-old mice, LRG protein levels were higher in 48-week-old mice. (B) The mean mLRG intensity in the 48-week-old mice was significantly increased compared with that in the 4- and 8-week-old mice as determined by one-way ANOVA followed by Dunnett’s test, **p < 0.001, Bars + SD. (C) Only weak LRG immunoreactivity was observed in the neurons and glial cells of 4- and 8-week-old mice, whereas significant LRG immunoreactivity was found in the neurons and glial cells of 48- and 108-week-old mice (I). Scale bar = 10µm. (D) Macroscopic findings of brain from 8-week-old mice. Scale bar = 5 mm. (E) Hematoxylin and eosin (HE) and Kluver-Barrera (KB) stains of coronal sections from 8-week-old mice and (F) 48-week-old mice. Scale bar = 500µm. (G) LRG proteins were significantly higher in Tg mice than in WT. (H) Densitometry analysis shows that LRG levels are approximately 2-to-4-fold higher in Tg mice, as determined by Mann-Whitney U test; *p < 0.05, **p < 0.001, Bars, + SD. (I) The cerebral cortex of 8-week-old and 48-week-old Tg mice presented significantly greater LRG immunoreactivity in the cytoplasm of neurons and glial cells than that of WT mice at 8 weeks and 48 weeks. Scale bar = 10µm. (J) Cerebral cortex of the 8- and 48-week-old Tg mice exhibited more LRG mRNA in the cytoplasm of neurons and glial cells than that of 8- and 48-week-old WT mice. Each red dot indicates one mRNA copy. Scale bar = 10µm. WT = wild type, Tg = transgenic.

Western blot analysis demonstrated that mLRG protein levels in 48-week-old mice (n = 5) were higher than those in 4- (n = 5) and 8-week-old mice (n = 5) (Figure 3A,B). Comparison of mLRG immunostaining of mouse cerebral cortex at 4, 8, 48, and 108 (n = 3) weeks of age confirmed only weak mLRG immunoreactivity in the neurons and glial cells of 4- and 8-week-old mice, while significant mLRG immunoreactivity was observed in the neurons and glial cells of 48- and 108-week-old mice (Figure 3C, I). In addition, mLRG in situ hybridization demonstrated that the cerebral cortex of 48-week-old mice displayed slightly increased mLRG mRNA expression compared to that of 8-week-old mice (Figure 3J). Thus, similar to human brain, mLRG expression was observed in the neurons and glial cells of mouse cerebral cortex and increased with age.

### Conditional Tg mice

We generated Tg mice that overexpressed mLRG in the neurons and glial cells of the brain. In 8-week-old Tg mice, macroscopic comparison with wild type (WT) showed that although the brain was smaller, there were no noticeable morphological changes in the cerebellum or brain stem (Figure 3D). Hematoxylin and eosin (HE) and Kluver-Barrera (KB) staining of coronal sections did not reveal any morphological abnormalities in the Tg brain (Figure 3E). In 48-week-old mice, we noted more severe thinning of the cerebral cortex, fewer granule cells, and moderate ventricular dilatation (Figure 3F). Western blot analysis confirmed that compared to WT, mLRG proteins were significantly higher in Tg mice (Figure 3G,H). Histologically, the cerebral cortex of 8- (n = 5) and 48-week-old Tg mice (n = 3) presented much more significant mLRG immunoreactivity and higher mRNA levels in the cytoplasm of neurons and glial cells than 8- (n = 5) and 48-week-old WT mice (n = 5) (Figure 3C,I, J).

**Figure 4:**
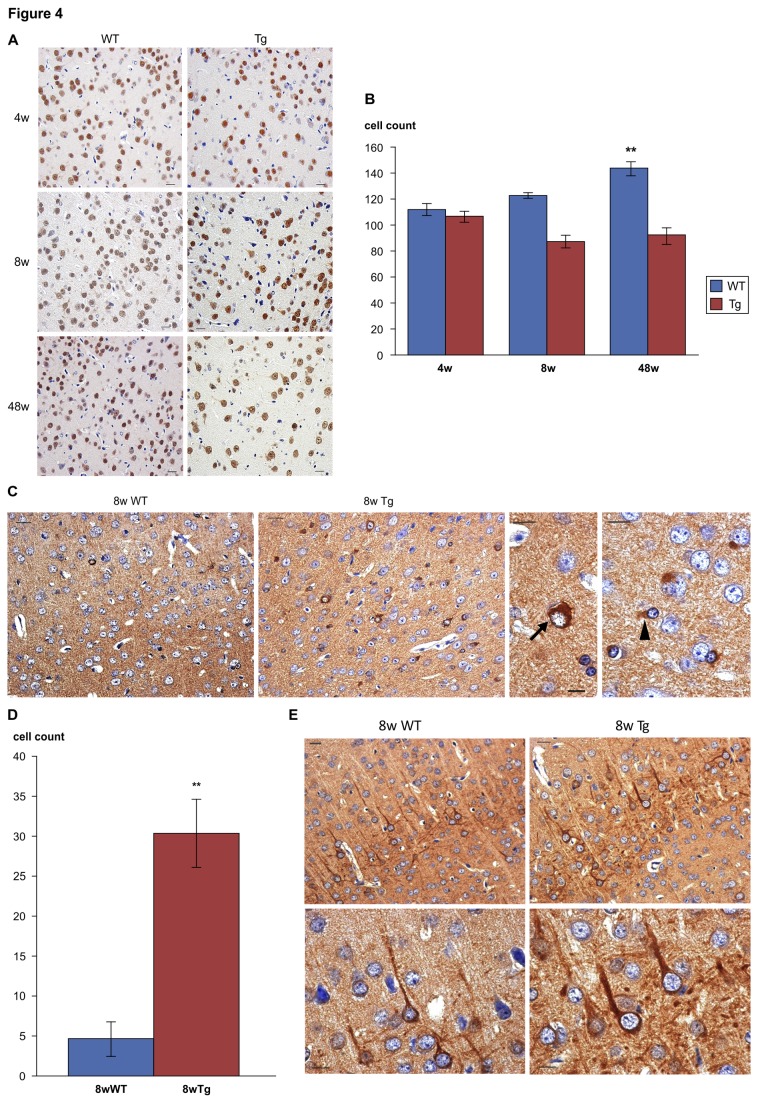
Immunostaining results for LRG transgenic mice. (A) Immunostaining of the cerebral cortex of 4-, 8-, and 48-week old mice using NeuN antibody. 4-week-old WT mice, 4-week-old Tg mice, 8-week-old WT mice, 8-week-old Tg mice, 48-week-old WT mice, and 48-week-old Tg mice. Scale bar = 20µm. (B) A difference in the number of NeuN-positive cells was already observed between 4-week-old Tg and WT mice, but with 8- and 48-week old mice, there was a significant decline in the number of NeuN-positive cells in Tg mice compared to that in WT as determined by Mann-Whitney U test, **p < 0.001, Bars, + SD. (C) Cortical immunostaining for phosphorylated tau in 8-week-old WT, 8-week-old Tg, Tg mice neurons (lower panel, arrow), and Tg mice glial cells (lower panel, arrow head). Scale bar = 20µm (upper panel) and 10µm (lower panel). (D) Tg cerebral cortex demonstrated significantly more neurons and glial cells that were positive for phosphorylated tau than WT as determined by Mann-Whitney U test, **p < 0.001, Bars + SD. (E) NF-L immunostaining in the cortex of 8-week-old WT mice and the cortex of 8-week-old Tg mice. Large, winding dendritic neurons in Tg mice suggested that neurodegeneration had occurred. Scale bar = 20µm (upper panel) and 10µm (lower panel). WT = wild type, Tg = transgenic.

We conducted immunostaining of cerebral cortex sections from 4-(WT, n = 5; Tg, n = 5), 8-(WT, n = 5; Tg, n = 5), and 48-week-old mice (WT, n = 5; Tg, n = 3) using NeuN antibody, which specifically stains neuronal nuclei, and then measured the number of cells demonstrating positive immunoreactivity. We did not observe a difference between Tg and WT mice regarding the number of NeuN-positive cells in 4-week-old mice, but there was a significant decline in the number of NeuN-positive cells in 8- and 48-week-old Tg mice compared to that in WT (Figure 4A,B).

Immunostaining for p-tau showed that the cerebral cortex of 8-week-old Tg mice demonstrated more positive neurons and glial cells than that of WT mice (Figure 4C,D). Furthermore, NF-L immunostaining demonstrated that 8-week-old Tg mice had large, winding dendritic neurons, which suggested that neurodegeneration had occurred (Figure 4E). In 8-week-old Tg mice, we confirmed neuronal decline and neurodegeneration seen in very old WT mice.

### hLRG protein interactors

Finally, we carried out a protein array to conduct a comprehensive screening of proteins bound with hLRG proteins. Analysis of the data for the proteins being studied relative to the negative control identified four candidate interactors for DYKDDDDK-hLRG at the 5 ng/µl concentration and 17 candidate interactors for DYKDDDDK-hLRG at the 50 ng/µl concentration (Table 3). Concentration-dependent signals were also observed for most candidate interactors identified at the 5 ng/µl concentration. The results include proteins identified using the statistical thresholds as described in the methods. We identified 17 proteins with an affinity factor of 3-fold or more. Of these, the affinities of presynaptic cytomatrix protein (PCLO), xin actin-binding repeat containing 2 (CMYA3), cortactin (CTTN), and peroxisomal trans-2-enoyll-CoA reductase (PECR) were dose dependent.

**Table 3:** Proteins that interact with hLRG.

**Database ID**	**Signal. Used**	**Neg Signal. Used**	**Ratio to Neg**	**Description**
BC001304.1	64880	540	120.1	piccolo (presynaptic cytomatrix protein) (PCLO)
BC022888.1	61327	668	91.9	xin actin-binding repeat containing 2 (CMYA3)
PV3359	62924	2078	30.3	Ephrin receptor A3 (EPHA3), transcript variant 1
BC009779.1	64257	2360	27.2	outer dense fiber of sperm tails 2-like (ODF2L)
BC054892.1	16653	940	17.7	dynein, light chain, roadblock-type 2 (DYNLRB2)
BC022054.1	8122	472	17.2	chromosome 10 open reading frame 83 (C10orf83)
NM_003929.1	20823	1446	14.4	RAB7, member RAS oncogene family-like 1 (RAB7L1)
PHC1244	10228	745	13.7	chemokine (C-C motif) ligand 19 (CCL19)
NM_197962.1	37044	3387	10.9	glutaredoxin 2 (GLRX2), transcript variant 2
NM_138565.1	65363	6544	10.0	cortactin (CTTN), transcript variant 2
NM_002867.2	6866	733	9.4	RAB3B, member RAS oncogene family (RAB3B)
NM_005335.3	7842	998	7.9	Hematopoietic lineage cell-specific protein
NM_021218.1	9660	2177	4.4	chromosome 9 open reading frame 80 (C9orf80)
NM_003720.1	6842	1909	3.6	Proteasome assembly chaperone 1
NM_030571.2	18949	5540	3.4	Nedd4 family interacting protein 1 (NDFIP1)
NM_018441.2	6965	2043	3.4	peroxisomal trans-2-enoyl-CoA reductase (PECR)
BC030592.2	12037	3933	3.1	HIV-1 Rev binding protein (HRB)

The data presented include the background subtracted pixel intensity value (the “Signal Used” value) and the ratio of Signal Used/Negative control Signal Used, which was used to rank the proteins.

We identified four candidate interactors for Flag-hLRG at the 5 ng/µl concentration and seventeen candidate interactors for Flag-hLRG at the 50 ng/µl concentrations. Concentration-dependent signals were observed for most candidate interactors identified at the 5 ng/µl concentration. The results include proteins identified using the statistical thresholds as described in the methods.

## Discussion

In the initial proteome analysis of CSF, we focused on LRG as a protein that is known to increase during iNPH [23,24]. Based on our results regarding LRG concentration in the CSF and immunoreactivity in the cerebral cortex, it became clear that LRG expression increases as a function of age [24]. Furthermore, as LRG is induced by inflammation, the observed increase with aging may actually be due to accumulated inflammation. We found that CSF hLRG concentrations were negatively correlated with the MMSE score in the NC and iNPH groups. This indicates that patients with dementia had higher hLRG concentrations than the non-demented patients.

As the CSF from PDD/DLB and PSP patients demonstrated higher LRG concentrations than the CSF from NC, iNPH, and AD patients, LRG may be an effective biomarker for identifying these diseases. In particular, it has been reported that PD, PSP, and AD demonstrate frequent comorbidity with iNPH; neurodegenerative disease is suspected in cases of iNPH with extremely high CSF concentrations of LRG [16].

In examining LRG levels in mouse cerebral cortex, it was clear that it increased with aging, similar to that in humans. To elucidate tLRG function, we constructed a Tg mouse that overexpressed mLRG in glial cells and neurons. Compared to WT, the brains of 8-week-old Tg mice were small but did not show any morphological abnormalities of the cerebellum or brain stem. However, in 48-week-old Tg mice, the cerebral cortex was thinner and the number of neurons in the cerebrum was significantly decreased.

LRG’s function may be related to its LRR structure. Leucine-rich repeat kinase 2 (LRRK2) possesses the same LRR structure as LRG. At present, mutations in LRRK2 represent the most common cause of late-onset familial PD and may be central to pathogenetic pathways for PD [25-33]. Although the functions of many of the members of the LRR-containing superfamily are known, the functions of LRG and LRRK2 have not been elucidated. Recently, both LRRK2 and LRG have been linked to Crohn’s disease (CD) [34,35]. Microglial activation is commonly observed in postmortem PD brain tissue, suggesting that inflammation may play a role in disease pathogenesis [36], although it is unclear whether this inflammation is causative or a secondary effect of earlier pathological events. Inflammation appears to be a common theme in PD and CD. Indeed, recent literature describes the roles for LRG and LRRK2 in immune response pathways [37-43].

Leucine-rich repeat transmembrane neuronal proteins (LRRTMs) also possess the same LRR structure as LRG. LRRTMs were recently found to guide presynaptic and mediate postsynaptic glutamatergic differentiation[44]. . Siddiqui [45] reported that LRRTMs bind neurexins with a differential code to cooperate in glutamate synapse development.

LRG function is thought to be mediated via binding with other proteins such as cytochrome *c* that restricts the functions of these proteins [14].

The protein microarray results indicated a strong affinity with Piccolo (PCLO), which is a novel component of the presynaptic cytoskeletal matrix (PCM) that is assembled at the active zone of neurotransmitter release. In addition, LRG demonstrates a strong affinity with cortactin, which exists in neuronal dendrites [46,47]. We speculated that strong binding of LRG with these proteins impedes synaptic function, reduces cognitive function, and is related to neuronal loss. Whether this observation is of potential physiological significance warrants further investigation.

## Conclusion

Our results show that in both humans and mouse brains, LRG increases with age. In humans, cognitive function declines with greater CSF LRG concentration. High CSF LRG concentrations were observed in both PDD/DLB and PSP, and this would be particularly useful in identifying patients with iNPH. Increased LRG in neurons and glial cells was associated with mouse cerebral cortex neurodegeneration. Due to the LRR structure of LRG, there is a possibility that it binds with proteins involved in synaptic function. However, these results needed to be validated in a different, and hopefully even larger, cohort of patients, preferably from an independent group.
